# Role of a *Bacillus subtilis* Direct-Fed Microbial on Digesta Viscosity, Bacterial Translocation, and Bone Mineralization in Turkey Poults Fed with a Rye-Based Diet

**DOI:** 10.3389/fvets.2014.00026

**Published:** 2014-12-16

**Authors:** Juan D. Latorre, Xochitl Hernandez-Velasco, Michael H. Kogut, Jose L. Vicente, Ross Wolfenden, Amanda Wolfenden, Billy M. Hargis, Vivek A. Kuttappan, Guillermo Tellez

**Affiliations:** ^1^Department of Poultry Science, University of Arkansas, Fayetteville, AR, USA; ^2^Facultad de Medicina Veterinaria y Zootecnia, Universidad Nacional Autónoma de México, Mexico City, Mexico; ^3^United States Department of Agriculture – Agricultural Research Service, Southern Plains Agricultural Research Center, College Station, TX, USA; ^4^Pacific Vet Group-USA, Inc., Fayetteville, AR, USA

**Keywords:** *Bacillus subtilis*, digesta viscosity, bone mineralization, turkey poults, rye

## Abstract

Rye contains high concentrations of non-starch polysaccharides (NSPs), leading to reduced digestibility. Since poultry have little or no endogenous enzymes capable of hydrolyzing these NSP, exogenous carbohydrases as feed additives are used in an attempt to reduce the anti-nutritional effects of these polysaccharides. Previously, an *in vitro* study conducted in our laboratory showed that inclusion of certain *Bacillus* direct-fed microbial (DFM) candidates that produce exogenous phytase, lipase, protease, cellulase, and xylanase in high-NSP diets significantly reduced both digesta viscosity and *Clostridium perfringens* proliferation. In the present study, rye-based turkey starter diets with or without *Bacillus*-DFM were administered *ad libitum* to day-of-hatch turkey poults in two independent experiments. In both experiments, day-of-hatch turkey poults were randomly assigned to either a control diet (CON) or a DFM treated diet (*n* = 25 birds/group). At 10 days-of-age, all turkey poults from experiments 1 and 2 were weighted and 12 turkey poults/group were randomly selected and humanely killed. Liver samples were aseptically collected to evaluate bacterial translocation, and intestinal digesta samples were individually collected to evaluate viscosity. Additionally, in experiment 2 both tibias were removed for assessment of bone parameters. In both experiments, the treated group showed a reduction in the total number of coliforms in the liver and a reduced digesta viscosity when compared to the CON group (*P* < 0.05). Turkey poults fed the *Bacillus*-DFM candidate had increased tibia diameter, breaking strength, ash content, calcium content, and phosphorus content when compared with CON turkey poults. In summary, turkey poults fed with a rye-based diet without DFM showed an increase in bacterial translocation and digesta viscosity, accompanied by a reduction in bone mineralization; however, these adverse effects can be prevented by the inclusion of selected a *Bacillus*-DFM candidate in high-NSP diets.

## Introduction

Maize is usually the main energy source in poultry diets, but in some countries, at times, it is difficult to formulate least cost diets using this cereal. In addition, the unpredictable price of maize suggests that the global poultry industry will continue to seek alternative cost-effective feed ingredients such as cereal by-products from biofuel and milling industries. Nevertheless, the successful inclusion of alternative ingredients in poultry diets will depend on the characterization of the nutritive value of these feed ingredients. Additionally, there is an increasing interest for technologies that can mitigate problems associated with anti-nutritional factors such as non-starch polysaccharides (NSPs) and phytates in poultry feed ingredients considering that monogastrics lack the endogenous enzymes necessary for digesting these components (1–5). Rye has been recognized as one of the cereals with the highest negative impact on performance parameters when included in poultry diets. It has been well documented that the high concentration of soluble NSP in rye increases digesta viscosity and stickiness of droppings, which results in poor performance (6–8). Furthermore, malabsorption of lipids and fat-soluble vitamins, deterioration of bone mineralization, and reduced leg soundness are all associated with rye utilization in poultry feeds (9). Soluble NSP in rye mainly comprised highly branched arabinoxylans, increasing digesta viscosity being responsible for poor digestibility through interference with the movement of particles and solutes across the intestinal lumen, preventing the access of digestive enzymes to the endosperm contents, and reducing intestinal absorption of sodium and calcium (10). Increased digesta viscosity reduces conjugated bile acids, affecting fat emulsification and fat digestibility (11). Recent studies published by our laboratory have shown that the significant reduction in bone strength and mineralization in chickens fed with rye (12), or gluten intolerance in human beings (13, 14), are also associated with malabsorption of minerals and fat-soluble vitamins. Since poultry has no enzymes capable of hydrolyzing NSP, exogenous xylanases are used as feed additives in an attempt to reduce the effect of this anti-nutritive factor (2, 4, 7). Previously, we have evaluated the inclusion of a selected *Bacillus* direct-fed microbial (DFM) candidate that produces exogenous enzymes (protease, phytase, lipase, xylanase, and cellulase) in high-NSP diets (rye, wheat, barley, and oat). In those studies, a significant reduction in both viscosity and *Clostridium perfringens* proliferation was observed between high-NSP control non-treated diets and the same diets supplemented with *Bacillus*-DFM *in vitro* (15, 16). In addition, studies conducted in broiler chickens using rye as an energy source in our laboratories also have shown that rye increased digesta viscosity, bacterial translocation, and leakage of fluorescein isothiocyanate dextran (FITC-d), altering the microbiota composition as well as bone mineralization in chickens (12). The objective of the present study was to evaluate the role of a *Bacillus subtilis*-based DFM candidate, which was selected for enzyme production on digesta viscosity, bacterial translocation, and bone mineralization in turkey poults fed with a rye-based diet.

## Materials and Methods

### Animal source and diets

In order to show that similar results can be achieved independently, two experiments were conducted in the present study. In each experiment, 50 day-of-hatch turkey poults were obtained from a commercial hatchery (Cargill, Gentry, AR, USA), and placed in isolators chambers with a controlled age-appropriate environment and *ad libitum* access to feed and water for 10 days. The number of animals used was based on published studies in which similar outcomes were measured (17–21). Turkey poults were randomly assigned to either a control group with a rye-based diet meeting the nutritional requirements for turkey poults as recommended by the National Research Council (22), or a treated group (TRT) fed with a rye-based diet supplemented with 10^6^ spores/g of feed of a specifically selected *Bacillus*-DFM candidate. No antibiotics were added to any of the diets (Table 1). All animal handling procedures were in compliance with Institutional Animal Care and Use Committee (IACUC) at the University of Arkansas, Fayetteville. Specifically, the IACUC approved this study under the protocol #11047 – “evaluation of direct-fed microbials and prebiotics in poultry.”

**Table 1:** **Composition of the experimental diets (g/kg)**.

Diet	Rye-based diet
Rye (126 g/kg CP)	372.4
Soybean meal (474.2 g/kg CP)	482.2
Poultry oil	79.5
Dicalcium phosphate	36.6
Ground limestone	11.3
Sodium chloride	4.1
dl-methionine	4.3
Vitamin premix^a^	1.0
l-Lysine HCl	5.0
Choline chloride 60%	1.0
Mineral premix^b^	1.0
Threonine	1.2
Selenium	0.2
Antioxidant^c^	0.2
**Calculated analysis**
ME, MJ/kg	11.9
Crude protein (g/kg)	285
Crude fat (g/kg)	96.2
Calcium (g/kg)	14.9
Total phosphorus (g/kg)	10.9
Lysine (g/kg)	18.2
Methionine (g/kg)	7.9
Methionine + cystine (g/kg)	11.8

*^a^Vitamin premix supplied per kilogram of diet: retinol, 9.2 mg; cholecalciferol, 100 μg; dl-α-tocopherol, 90 mg; menadione, 6 mg; thiamine, 6.2 mg; riboflavin, 26.5 mg; pantothenic acid, 39.7 mg; niacin, 100 mg; pyridoxine, 11 mg; folic acid, 4 mg; biotin, 0.3 mg; cyanocobalamin, 0.1 mg*.

*^b^Mineral premix supplied per kilogram of diet: Mn, 70 mg; Zn, 40 mg; Fe, 37 mg; Cu, 6 mg; I, 0.7 mg; Co, 0.2 mg*.

*^c^Ethoxyquin*.

### DFM preparation

In an effort to grow high numbers of viable spores, a solid state fermentation media (SS) developed by Zhao et al. (23) was selected and modified for use in these experiments. Briefly, a liquid media component was added to a mixture of 70% rice straw and 30% wheat bran at a rate of 40% by weight. The SS media was added to a 250 ml Erlenmeyer flask and sterilized by autoclaving for 30 min at 121°C. Candidate isolates were grown individually overnight at 37°C in TSB, then 2 ml of a candidate culture were added to the prepared SS media. The inoculated flasks were incubated for 24 h at 37°C and then incubated for another 72 h at 30°C. The cultures were removed from their flasks, placed onto petri dishes, and then dried at 60°C. Following this, the cultures were aseptically ground into a fine powder to generate stable spores (~10^11^ spores/g). Spores were mixed into the feed using a rotary mixer for 15 min. Samples of feed containing the *Bacillus*-DFM culture were taken and a 1:10 dilution was made with saline. All samples were subject to 100°C for 10 min, enumerated using 10-fold dilutions and plate-counting following overnight incubation at 37°C on tryptic soy agar plates (TSA, catalog no. 211822, Becton Dickinson, Sparks, MD, USA) (Data not shown).

### Experimental design

In experiments 1 and 2, 50 day-of-hatch, turkey poults were randomly assigned to 1 of 2 groups (*n* = 25). At 10 days-of-age, all turkey poults in both experiments were weighed and 12 turkey poults were randomly selected and humanely killed using carbon monoxide asphyxiation method. The right half of the liver was collected aseptically to evaluate bacterial translocation. Additionally, digesta samples were individually collected to evaluate viscosity and, and in experiment 2, both tibias were used to analyze bone parameters as described below.

### Viscosity

Total intestinal contents were collected from Meckel’s diverticulum to the ileocecocolonic junction. For viscosity analysis, ~1.5 g (wet weight) of the fresh digesta were immediately centrifuged (12,000 × *g*) for 5 min. The supernatant was collected and stored on ice until viscosity was determined using a LVDV-I Brookfield digital cone-plate viscometer fitted with a CP-40 spindle (Brookfield Engineering, Middleboro, MA, USA). The analyzed samples and the viscometer cup were maintained at a temperature of 40°C during viscosity measurement. Viscosity was measured in centipoise (cP = 1/100 dyne s/cm^2^) and the results were reported as log_10_ cP.

### Bacterial translocation

Briefly, the right half of the liver was removed from each poult, collected into sterile bags, homogenized, weighed, and 1:4 wt/vol dilutions were made with sterile 0.9% saline. Ten-fold dilutions of each sample were made in a sterile 96 well Bacti flat bottom plate, and the diluted samples were plated on MacConkey Agar (VWR Cat. No. 89429-342, Suwanee, GA 30024, USA). Biochemical evaluation tests as well as identification of isolated colonies were carried out using a bioMerieux API-20Etest kit (catalog no. 20100, bioMerieux, Marcy l’Etoile, France). BT was expressed in colony forming units (Log_10_ cfu/g of tissue).

### Bone parameters

In experiment 2, bone parameters were measured according to the methods described by Zhang and Coon (18). Tibias from each poult were cleaned of attached tissues. Bones from the left leg were subjected to conventional bone assays as described below, and the tibias from the right legs were used to determine breaking strength. The bones from the left tibia were dried at 100°C for 24 h and again weighed. Then the samples were ashed in a muffle furnace (Isotemp muffle furnace, Fisher Scientific, Pittsburgh, PA, USA) at 600°C for 24 h in crucibles, cooled in a desiccator, and weighed. Finally, the content of calcium and phosphorus in the tibia was determined using standard methods (24). The right tibial diaphyses from individual birds were cleaned of adherent tissues, the periosteum was removed, and the biomechanical strength of each bone was measured using an Instron 4502 (Norwood, MA, USA) material testing machine with a 509 kg load cell. The bones were held in identical positions and the mid-diaphyseal diameter of the bone at the site of impact was measured using a dial caliper. The maximum load at failure was determined using a three-point flexural bend fixture with a total distance of 30 mm between the two lower supporting ends. The load, defined as force in kg/mm^2^ of cross-sectional area (kg/mm^2^), represents bone strength. The rate of loading was kept constant at 20 mm/min collecting 10 data points per second using Instron’s Series IX Software (Norwood, MA, USA).

### Statistical analysis

All data were subjected to one-way analysis of variance as a completely randomized design using the General Linear Models procedure of SAS (25). Data are expressed as mean ± SE. A *P*-value of *P* < 0.05 was set as the standard for significance.

## Results and Discussion

Thousands of years of evolution shaped the digestive system of the jungle fowl to deal with the dietary ingredients they encounter in an efficient manner. More recently, through intensive genetic manipulation, nutrition, and health programs, we have altered the biology and growth potential of poultry among other productive animals (26, 27). In the wild, the diets of these animals would be made up of many different ingredients, few of which would ever reach more than 30% of total intake on a lifetime basis. The range in types and relative quantities of ingredients that can be presented to the modern commercial monogastric animals, while complex, tend to result in a diet in which two or three ingredients may constitute around 75% of intake (22). Such change is driven by least cost formulation processes, and endeavors to provide maximum nutrient density for minimum cost (7). Maize is usually the main source of energy in poultry diets, but at times it is difficult to formulate least cost diets using maize and unconventional grains have to be used. When poultry are fed alternative grains such wheat or rye that are high in NSPs, poor performance, and unmanageable litter conditions caused by sticky droppings are reported (6, 10, 17). Wheat or rye contains high concentrations of NSP, leading to reduced digestibility. In addition, high-NSP diets have also been associated with necrotic enteritis, a multi-factorial disease caused by *C. perfringens* that is probably the most important bacterial disease in terms of economic implications in broiler chickens (28–30). Therefore, feeding enzymes for swine and poultry have made the largest impact in the past decade to solve problems associated with grains rich in NSP (7). NSP-degrading enzymes (NSPases, xylanase, β-glucanase, β-mannanase, α-galactosidase, and pectinase) improve nutrient digestibility and reduce digesta viscosity (6, 31). In the present study, a significant improvement in body weight by day 10-of-age was observed in turkey poults fed rye with added *Bacillus*-DFM candidate when compared to turkey poults that were fed an unsupplemented rye-based diet (*P* < 0.05). Turkey poults from the TRT group in both experiments showed a significant reduction in digesta viscosity, which was associated with a decrease in bacterial translocation to the liver (Table 2). The lactose positive colonies obtained from liver samples were identified as *E. coli*. Feeding diets that are high in NSP and high viscosity may be the pathological mechanism underlying bacterial translocation of gut microflora from the intestinal lumen, which predisposes poultry to systemic bacterial infections (32–35). Inflammatory responses to gut-derived and blood-borne pathogens typically occur in the liver and spleen, which are the major organs that remove bacteria and toxins, including lipopolysaccharides (LPS), from the bloodstream (36). Particularly, the levels of LPS, a component of Gram-negative bacteria, are increased in the portal and/or systemic circulation with impaired gut epithelial integrity and dysbacteriosis (37). Strong evidence suggests that pathogen-derived compounds from the gut have a major role and modulating effect on liver diseases and chronic inflammation (38, 39). Diet ingredients, integrity of the gut epithelium, immune defense in the gut and in the liver, as well as the composition of the microbiome in the intestinal tract all appear to play an integrated role in the maintenance of health and balance in the gut–liver axis (40). The results of the present study suggest that rye-based diets can both enhance bacterial translocation and digesta viscosity, but these adverse effects can be prevented by the inclusion of this specific *Bacillus*-DFM candidate (Table 2).

**Table 2:** **Evaluation of body weight, digesta viscosity, and bacterial translocation to the liver in neonatal turkey poults fed with a rye-soybean based diet with or without dietary inclusion of a *Bacillus* direct-fed microbial (DFM) in experiment 1 and 2**.

	Experiment 1			Experiment 2		
	Body weight (g)	Digesta viscosity (cP Log_10_)	Bacterial translocation (cfu Log_10_)	Body weight (g)	Digesta viscosity (cP Log_10_)	Bacterial translocation (cfu Log_10_)
CON^c^	65.91 ± 3.61^b^	2.03 ± 0.31^a^	3.03 ± 0.51^a^	74.47 ± 1.59^b^	2.80 ± 0.45^a^	2.13 ± 0.67^a^
TRT^d^	82.85 ± 4.23^a^	1.54 ± 0.22^b^	1.24 ± 0.51^b^	95.60 ± 2.17^a^	1.62 ± 0.53^b^	0.35 ± 0.40^b^

*^a,b^Superscripts within columns indicate significant difference at *P* < 0.05*.

*^c^Control rye-based diet*.

*^d^Control rye-based diet with candidate *Bacillus*-DFM*.

*Body weight *n* = 25; intestinal viscosity and bacterial translocation *n* = 12. Data are express as mean ± SE*.

*Intestinal viscosity is expressed in Log_10_ (in centipoise, cP = 1/100 dyne s/cm^2^)*.

*Liver bacterial translocation (expressed in cfu Log_10_/g of tissue)*.

Bone parameters were measured in experiment 2 to determine whether the addition of the specifically selected *Bacillus*-DFM candidates would counteract the loss of mineral and vitamin utilization that occurs when feeding a rye-based diet. There was a significant increase (*P* < 0.05) in tibial diameter, tibial breaking strength, as well as the ash, calcium, and phosphorus content of the tibias observed when the selected candidate DFM was added to the rye-based diet in comparison to rye-based diet fed turkey poults without the *Bacillus*-DFM (Table 3). The significant reduction in bone mineralization observed in the control group confirmed previous studies that have shown that high-NSP diets in poultry or gluten intolerance in human beings is also associated with deterioration of bone mineralization and leg soundness (13, 14, 17, 41–44). In the present study, dietary supplementation of a selective DFM candidate, which has the ability to secrete exogenous enzymes (xylanase, cellulose, protease, phytase, and lipase) was able to significantly improve performance, decrease digesta viscosity, and improve bone mineralization (experiment 2), suggesting that the possibility of using this feed additive as an alternative to feed enzymes. These results also confirm in some extent our earlier findings that have shown significant reduction in both viscosity and *C. perfringens* proliferation between high-NSP control non-treated diets or the same diets supplemented with the selective DFM candidate *in vitro* (12, 15, 16). Together, they represent a step toward the application of nutrigenomics in the context of a poultry model. The incorporation of one or more “omics” techniques (in particular, assessment of the microbiome) will provide a better understanding of how dietary food components can affect physiological functions and the fundamental cellular and molecular mechanisms implicated in the digestive process of high-NSPs diets in poultry.

**Table 3:** **Evaluation of bone strength and bone composition in neonatal turkey poults fed with a rye-soy based diet with or without dietary inclusion of a *Bacillus* direct-fed microbial (DFM) in experiment 2**.

	Tibia strength load at yield (kg/mm^2^)	Tibia diameter (mm)	Total ash from tibia (%)	Calcium (% of ash)	Phosphorus (% of ash)
CON^c^	0.26 ± 0.02^a^	4.45 ± 0.32^a^	35.61 ± 0.81^a^	27.35 ± 0.07^a^	16.35 ± 0.52^a^
TRT^d^	0.44 ± 0.03^b^	5.82 ± 0.78^b^	50.87 ± 0.75^b^	40.31 ± 0.46^b^	22.67 ± 0.29^b^

*^a,b^Superscripts within columns indicate significant difference at *P* < 0.05*.

*^c^Control rye-based diet*.

*^d^Control rye-based diet with candidate *Bacillus*-DFM*.

*Tibias from 12 turkey poults were collected to evaluate bone qualities. Data are expressed as mean ± SE*.

In summary, the results of the present study confirm our previous investigation conducted in chickens fed with rye, which showed a significant increase in digesta viscosity that has been associated with low performance, increased enteric bacterial translocation to liver, and decreased bone strength (12). Large scale commercial studies to evaluate dietary inclusion of selected *Bacillus*-DFM candidates that produce exogenous enzymes in high-NSP diets in poultry, on performance parameters, microbiome composition, and incidence of necrotic enteritis caused by *C. perfringens* are currently being evaluated.

## Conflict of Interest Statement

The authors declare that the research was conducted in the absence of any commercial or financial relationships that could be construed as a potential conflict of interest.
